# Fabrication of a Micron-Scale Three-Dimensional Single Crystal Diamond Channel Using a Micro-Jet Water-Assisted Laser

**DOI:** 10.3390/ma14113006

**Published:** 2021-06-01

**Authors:** Qiang Wei, Xiaofan Zhang, Fang Lin, Ruozheng Wang, Genqiang Chen, Hong-Xing Wang

**Affiliations:** Key Laboratory of Physical Electronics and Devices of the Ministry of Education, School of Electronic Science and Engineering, Xi’an Jiaotong University, Xi’an 710049, China; wbgwei@xjtu.edu.cn (Q.W.); xiaofan.z@xjtu.edu.cn (X.Z.); leaf-lin@xjtu.edu.cn (F.L.); wangrz@xjtu.edu.cn (R.W.); genqiangchen@stu.xjtu.edu.cn (G.C.)

**Keywords:** single crystal diamond, micro-water jet guided laser, microchannel

## Abstract

Two types of a trench with conventional vertical and new reverse-V-shaped cross-sections were fabricated on single crystal diamond (SCD) substrate using a micro-jet water-assisted laser. In addition, a microwave plasma chemical vapor deposition device was used to produce multiple micrometer-sized channels using the epitaxial lateral overgrowth technique. Raman and SEM methods were applied to analyze both types of growth layer characterization. The hollowness of the microchannels was measured using an optical microscope. According to the findings, the epitaxial lateral overgrowth layer of the novel reverse-V-shaped trench produced improved SCD surface morphology and crystal quality.

## 1. Introduction

Infiltrated with ever-advancing power semiconductor devices in the third century, they played a significant role in daily life. The objective is to make power semiconductors faster, smaller, lighter, and durable, resulting in lower overall device costs. However, high-temperature operations require a lot of power. Therefore, the diamond semiconductor, one of the most promising materials in industrial applications, has received significant research attention in electronic process thermal management, because of its stable chemical properties, high-temperature operation, and low friction coefficient and low thermal expansion coefficient. In addition, diamond has an extremely hard-wearing resistance and thermal conductivity of 2000–2600 W/(m·K) [1,2].

Diamond with microchannels is an ideal substrate for cooling chip temperatures and achieving low thermal resistance [3,4,5]; enlarging surface and heat exchange areas of catalytic elements [6,7,8,9], and assisting with liquid transport as a stent in medical equipment [10,11]. Due to the wider application of diamond microchannels, the importance of improving microchannel fabrication has increased.

Ion beam lithography is the most commonly documented method of fabricating single-crystal diamond microchannels [12,13], and microstructures are prepared on diamond substrates by femtosecond laser technique [14,15]. At the same time, epitaxial lateral overgrowth techniques are adopted to form closed channels [16,17,18]. Cutting trenches using a perpendicular micro-jet water-guided laser as a feasible means of fabricating a single crystal diamond (SCD) microchannel has been reported [19]; however, the surface quality of this type of microchannel was not improved, and defects remained. Therefore, a new method of producing microchannels with improved performance and efficiency is needed.

This research work concerns the production of three-dimensional microchannels with large and controllable geometry on SCD substrate. The cross-section of the microchannel on SCD substrate had a reverse-V shape, which was machined using a micro-jet water-assisted laser. Then, epitaxial lateral overgrowth was applied using a microwave plasma chemical vapor deposition system on the processed substrate. An optical microscope and scanning electron microscope (SEM) were used to distinguish the differences in the growth area and identify growth layer morphology, and determine the channel hollowness. Raman and X-ray diffraction (XRD) techniques were applied for characterization and quality determination. Finally, the principle of growth is shown in illustrations schematically.

## 2. Experiment

**Figure 1:**
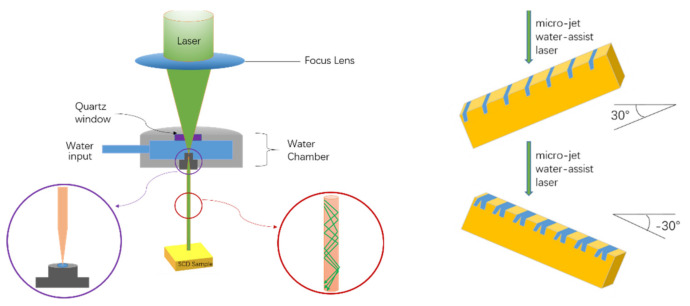
Schematic of the micro-jet water-assisted laser.

A high-pressure and high-temperature (HPHT) SCD (001) substrate was used as the experimental material, an Ib type monocrystalline diamond purchased by “Element 6” (Element Six UK Ltd., Oxfordshire, UK), with a deflection angle of 0.002° on its (001) surface. The full width at half maximum of the rocking curve in (004) direction is 0.015°, and lattice relaxation characteristics show in (311) direction, indicating that this HPHT substrate has good single crystal quality. Furthermore, it was found that there were obvious nitrogen-related optical characteristics in the monophonic region (1 of 400–1400 cm) by FTIR spectrum test (Nicolet iS50, Thermo Scientific, Waltham, MA, USA). Therefore, it accords with the characteristics of type Ib monocrystalline diamond [20,21], with dimensions of 3 × 3 × 1 mm^3^. The substrate was placed on the three-dimensional *X*-, *Y*-, and *Z*-axis removable workpiece stage of a laser system, which comprised a Nd:YAG (Neodymium Doped Yttrium Aluminum Garnet) laser (Lee Laser Inc., Orlando, FL, USA) and a 50 W high-power supply. The laser wavelength was 532 nm, and the focusing system consisted of quartz optical lenses; the water chamber was filled with ultra-pure de-ionized (DI) water (Elga, Altleiningen, Germany). The laser beam focused by focusing lens and completely reflected at the air-water interface in a manner similar to optical fiber. The protect gas was used to prolong micro-thin water jet, which guides the laser mean precisely and avoids beam divergent. The machining heat on cutting zone and debris is eliminated by water-jet and leaves a smooth kerf. The DI water pressure, nozzle diameter, and helium protect gas flow were 400 bar, 50 μm, and 1.1 L/min, respectively. The cutting speed was held constant at 8 mm/s based on a previous research result. The SCD substrate was treated by micro-jet water-assisted laser and rotated in certain degree as shown in Figure 1.

Two styles of the trench were fabricated. The first was a conventional trench, for which the laser was positioned perpendicular to the SCD substrate surface, resulting in an approximately rectangular cross-section. The other was an exploratory trench with a reverse-V-shaped cross-section. In detail, the diamond substrate was cut twice by the micro-jet water-assisted laser and rotated twice by a given number of degrees with a shared opening.

Because the inner face of the SCD substrate trench was carbonized by laser machining, hydrogen/oxygen plasma and a mixed acid of H_2_SO_4_ (Alfa Aesar, Waltham, MA, USA) and HNO_3_ (Alfa Aesar, Waltham, MA, USA) at 250 °C were applied for 3 h to clean the non-diamond phase. The growth conditions were as follows: 950 °C substrate temperature, 500 sccm of H_2_ flow, 40 sccm of CH_4_ flow, and gas pressure of 110 Torr. It took 10 h to obtain the desirable as-grown diamond layer and the thickness was about 100 μm. The epitaxial lateral overgrowth (ELO) method was applied to assist with merging the trench sidewalls and the formation of microchannels.

## 3. Results and Discussion

**Figure 2:**
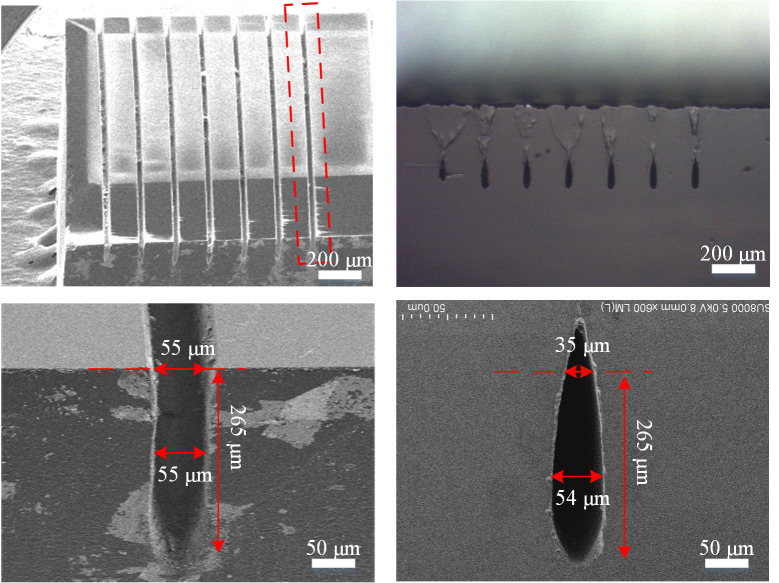
Cross-section SEM image of single crystal diamond (SCD) substrate (**a**) after laser treatment (45°); (**b**) after epitaxial lateral diamond overgrowth; (**c**) enlarged view of trenches; (**d**) enlarged view of (**b**).

The SEM images (Gemini SEM 500, Zeiss, Jena, Germany) of conventional perpendicular trenches in the diamond substrate after laser treatment are shown in Figure 2a, which shows that the trenches made by the traditional method are parallel, characterized by perfect uniformity with clearly visible borders. The morphology of each trench is identical, with trench length, top opening width, central width, and overall depth of 3 mm, 55 μm, 55 μm, and 265 μm, respectively, as shown in Figure 2c. The width of the fabricated trench indicates that the cross-section after laser treatment was an approximate rectangle shape with a curved bottom, as shown in Figure 2c. Figure 2b is a SEM image of the cross-section of the diamond substrate after epitaxial lateral overgrowth using the microwave plasma chemical vapor deposition system; the figure shows that the trench top is closed and fully covered by single crystal diamond using epitaxial overgrowth process.

After merging, growth occurred in horizontal and vertical directions on the entire surface of the substrate crystal, allowing a good quality SCD layer to be obtained. The SEM image of the enlarged trench cross-section after growth is shown in Figure 2d; notably, the top width decreased to 35 μm. However, the central width was maintained at 55 μm. Based on these observations, we calculated the opening width reduced by 36%. Furthermore, after 10 h of epitaxial lateral overgrowth, the sidewalls of each trench gradually merged into each other during horizontal and vertical growth, indicating the water drop-shaped microchannel was fabricated, and its overall depth was greater than the trench depth.

**Figure 3:**
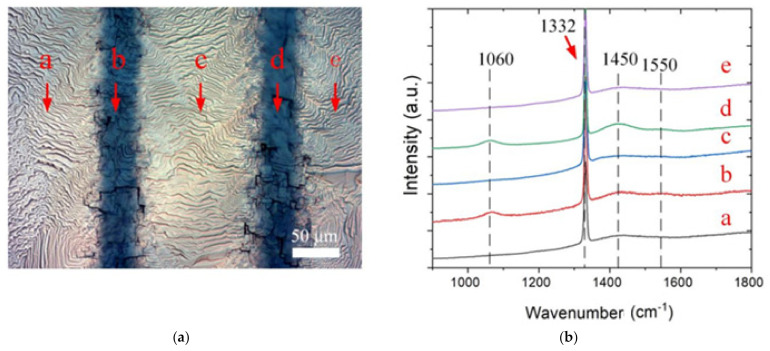
(**a**) After growth of the image; (**b**) Raman spectra taken from different locations around the channel (as shown in the adjacent images).

An optical microscope in reflection mode (STM7, Olympus Corporation, Tokyo, Japan) was used to explore the morphologies around the microchannel, as shown in Figure 3a, where the dark stripes represent microchannels and the bright area represents homoepitaxial growth. In addition, the Raman spectra (Laser Raman Spectrometer, Horiba Scientific, Kyoto, Japan) were collected from points (a–e) specified on the optical microscope picture of the substrate the following growth to analyze the diamond structure and phase composition along the channel.

The Raman excitation wavelength was 532 nm, which shows a strong peak at 1332 cm^−1^ in all five (a–e) regions, indicating a good composition of the single-crystal diamond [22,23], as shown in Figure 3b. In addition, a peak was located in the Raman spectrum at 1430–1470 cm^−1^, and there was a characteristic peak produced by transpolyacetylene [24], which often appears in CVD diamond. It could be seen that the transpolyacetylene peak at points a, b and c was extremely poor, while being significantly enhanced at positions b and d. In addition, a characteristic peak appeared at 1550 cm^−1^, indicating disordered carbon correlation [25,26]. The peak position coincided with the cutting groove area, which indicated that the graphitization on the diamond surface caused by laser was not eliminated in the pickling process. It is rare that the characteristic peak shows in 1060 cm^−1^ different from T peak, which is only seen in ultraviolet excitation [24]. The excitation spectrum around 1060 cm^−1^ was triggered by a kind of polyacetylene with a long molecular chain. Harada I et al. [27] mention that the molecular chain with energy bands close to the excitation source will be preferentially excited under different excitation energies. Gussoni et al. also recorded the peaks of Raman spectrum at 1070 and 1470 cm^−1^ by Raman transitions with higher excitation energies [28,29]. It is speculated that 1060 cm^−1^ is the characteristic peak of polyacetylene, corresponding to the peak at 1450 cm^−1^. Therefore, the conventional vertical trench after growth at positions b and d shows the characteristic peak at 1060 cm^−1^, revealing polyacetylene in diamond growth.

**Figure 4:**
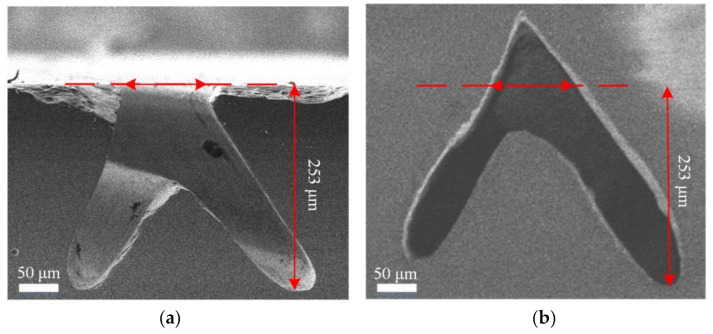
(**a**) SEM image of reverse-V-shaped microchannel; (**b**) After growth.

Figure 4a shows a schematic diagram of the formation of the reverse-V-shaped trench. The flat HPHT SCD substrate was rotated 30° to a vertical direction to fabricate the first tilted channel using the micro-jet water-guided laser and then rotated −30° to fabricate the second channel by sharing the top opening. Following the cutting procedure, the substrate was subjected to acid cleaning to remove all graphite residue and flaws left by the laser treatment. The growth of the substrate was carried out via epitaxial lateral overgrowth using a microwave plasma chemical vapor deposition system (6500, SEKI, Japan) under the same conditions as those used for the conventional perpendicular trenches. In the SEM image of the cross-section after growth, it is noticeable that the reverse-V-shaped trench opening completely disappeared, leaving a triangle shape and a closed surface free of any defects, as shown in Figure 4b. The top opening width decreased from 74 to 69 μm, indicating shrinkage of 7%, and lateral growth was effectively inhibited. The trench top gradually closed during the merger of horizontal and vertical growth, showing that the overall depth was greater than that before the growth, and all of the trenches formed a reverse-V-shaped microchannel.

**Figure 5:**
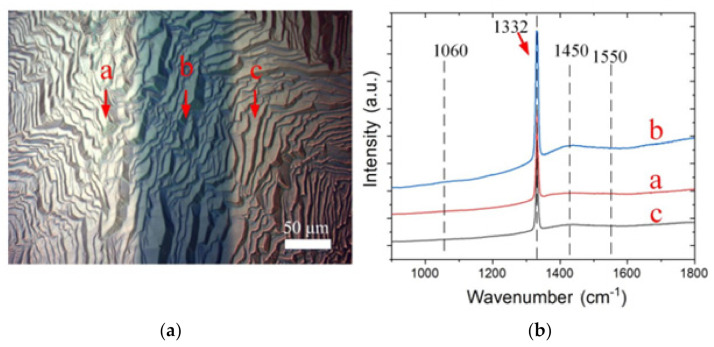
(**a**) SEM image after the growth; (**b**) Raman spectra taken from different locations around the channel (as shown in the adjacent images).

The surface optical microscope image of the reverse-V-shaped microchannel after growth is shown in Figure 5a, revealing a larger dark stripe than that of the conventional vertical channel. Both dark and light regions indicate a consistent morphology of single-crystal diamond characterization. Raman spectra at an excitation wavelength of 532 nm were applied to investigate the crystal composition around the microchannel, for which positions a–c are marked, as shown in Figure 5. It indicates a very poor transpolyacetylene peak at 1430–1470 cm^−1^, additionally to a strong peak at 1332 cm^−1^ in positions a and c. The transpolyacetylene peak and weak disordered carbon peak could still be measured at point b. Still, it was significantly lower than that in Figure 3b at 1060 cm^−1^, indicating that the cutting grooves of two structures affected the growth of epitaxy diamond crystals.

**Figure 6:**
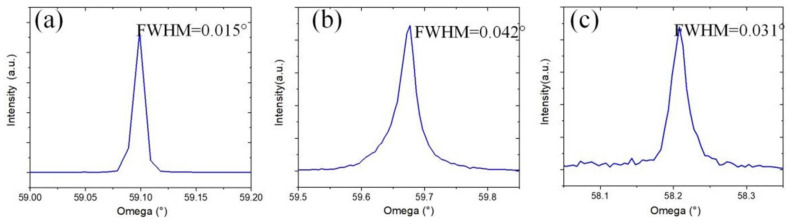
XRD scanning: (**a**) SCD substrate; (**b**) vertical microchannel; (**c**) reverse-V-shaped microchannel.

Types of microchannel Figure 6 show the results of XRD (X’pert Pro MRD, PANalytical, Kassel, Germany) examination following the growth. XRD analysis was conducted, as shown in Figure 6. The full width at half maximum value of substrate was 0.015° (Figure 6a), and those of the epitaxial lateral overgrowth film of the conventional vertical microchannel and the reverse-V-shaped microchannel were 0.042° and 0.031°, respectively; which are presented in Figure 6b,c. It is noticeable that the quality of the epitaxial lateral overgrowth value of the reverse-V-shaped cutting strategy is better than that of the conventional vertical microchannel, and is comparable to the substrate, indicating an acceptable quality.

**Figure 7:**
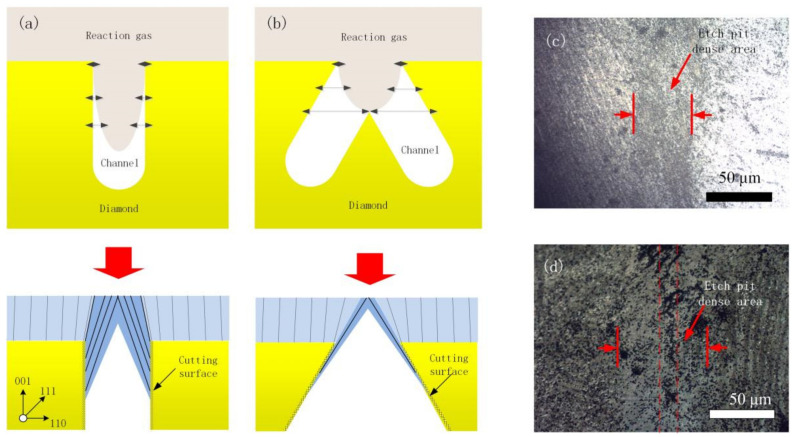
Two types of microchannel: (**a**) vertical trench; (**b**) reverse-V-shaped trench; (**c**) etch pit distribution of vertical trench; (**d**) etch pit distribution of reverse-V-shaped trench.

Finally, the principle of the growing difference of each type of microchannel was analyzed; schematic diagrams of the growth status of the conventional vertical trench and reverse-V-shaped trench are shown in Figure 7, respectively. During the growth stage, reaction gas penetrated the microchannel and interacted with the channel inter-wall of the single-crystal diamond. It is noticeable that the reaction gas concentration decreased with the increase in channel depth. About the ELO process, optimal growth of (110) or (111) surfaces was required to merge the channels. However, in the vertical cutting groove, there was a small amount of graphitized surface on the cutting surface due to the action of the diamond laser, with relatively large roughness. Besides, the quality of the diamond growing on the surface was significantly affected, and many dislocations were generated. As the diamond grew, the dislocation was transferred to its surface, as shown in Figure 7a. It can be seen from Figure 2d that the convergence of the sidewall started from the inside of the cutting, thus forming a contractionary teardrop-shaped cross-section channel.

The cross-section of the conventional vertical microchannel reveals a smaller cavity area than that of the reverse-V-shaped channel. Therefore, the concentrations of reaction gas in the inter-wall of the two types of the microchannel are significantly different. The growing environment during the SCD formation of the conventional vertical microchannel is more concentrated and expands inwards. The central and bottom areas of the reverse-V-shaped microchannel barely interact with the gas, so the growth rate of the sidewall is really slow, and only a few defects generated by the sidewall growth are transferred to the diamond surface by (111) surfaces [30,31]. As a result, the surface defects are reduced, resulting in slow groove shrinkage. As shown in Figure 7b, the two samples were etched by H_2_/O_2_ plasma. The 500 sccm flow rate of H_2_ and 5 sccm flow rate of O_2_ forming H_2_/O_2_ plasma were used to etch the diamond surface to express dislocation [32]. The cavity pressure, microwave power, temperature and time were 90 torr, 3000 W, 1000 °C and 60 min, respectively, an etching process. Figure 7c shows the etching pits on the surface of the vertical channel. It can be seen that the areas on both sides of the channel are relatively bright, which states that the density of etching pits is low; while in the groove area, it is significantly increased. However, in the area of reverse-V-shaped microchannel, the density of etching pits decreases significantly.

This is because most of the area is high-quality crystals produced by the lateral epitaxy on the surface. In contrast, in the center area of the groove, a relatively high defect shadow area with a width of about 10 μm appears, which also proves the rationality of the model in Figure 7b.

Because the channel inter-wall was roughened by amorphous diamond during laser cutting, flaws and defects appeared and affected the quality of lateral growth. The comparison shows that the reverse-V-shaped microchannel can improve the top surface quality of the microchannel and inhibit extended defects.

**Figure 8:**
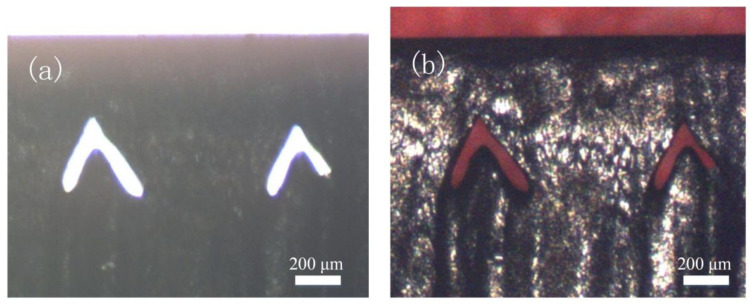
Cross-section optical microscope images of SCD microchannels with various backlights from (**a**) white light; (**b**) red light.

Figure 8a shows the optical microscope image of the reverse-V-shaped microchannel cross-section, for which the boundary can be observed under a white backlight. To determine the hollowness and continuity, as shown in Figure 8b, an optical microscope image of the microchannel cross-section was taken using a red backlight from the front and rear, which indicated that the microchannel was coherent and continuous.

## 4. Conclusions

In summary, reverse-V-shaped three-dimensional microchannels in single crystal diamond were fabricated. First, micro-trenches were fabricated using a micro-jet water-assisted laser cutting technique. This was followed by the fabrication of microchannels using an epitaxial lateral overgrowth technique. Optical microscope and SEM images showed the morphologies and structures of microchannel formation. Raman and XRD spectra were used to evaluate the characteristics of the crystal after growth and indicated an acceptable single-crystal diamond quality was achieved. Schematics were used to illustrate the advantages of the reverse-V-shaped microchannel in inhibiting diamond growth defects compared to the conventional vertical microchannel. The coherence and hollowness of the reverse-V-shaped microchannel were determined using backlights of two colors and indicated the microchannel’s consistency and continuity. This work could provide a controllable process to fabricate single-crystal diamond heat sinks for application in high-power devices.

## Data Availability

Data available on request due to restrictions, eg privacy or ethical.
